# Cigarette smoke extract enhances neutrophil elastase-induced IL-8 production via proteinase-activated receptor-2 upregulation in human bronchial epithelial cells

**DOI:** 10.1038/s12276-018-0114-1

**Published:** 2018-07-06

**Authors:** Kyoung-Hee Lee, Jinwoo Lee, Jiyeong Jeong, Jisu Woo, Chang-Hoon Lee, Chul-Gyu Yoo

**Affiliations:** 1 Division of Pulmonary and Critical Care Medicine, Department of Internal Medicine, Seoul National University Hospital, Seoul, 03080 Korea; 2 Department of Internal Medicine, Seoul National University College of Medicine, Seoul, 03080 Korea

**Keywords:** Mechanisms of disease, Cell signalling

## Abstract

Although inflammation, oxidative stress, and protease-antiprotease imbalance have been referred to as a pathogenic triad in chronic obstructive pulmonary disease (COPD), little is known about how they interact. The objectives of this study were to elucidate the effect of cigarette smoke extract (CSE) on the neutrophil elastase (NE)-induced inflammatory response and its molecular mechanism in bronchial epithelial cells. We observed that NE activated extracellular signal-regulated kinase (ERK) and induced IL-8 production. Blocking ERK activation using a MEK inhibitor (U0126) suppressed NE-induced IL-8 secretion and knockdown of proteinase-activated receptor 2 (PAR2) using siRNAs inhibited both NE-induced ERK activation and subsequent IL-8 release, suggesting that NE-induced IL-8 production is dependent on PAR2-mediated ERK activation. Interestingly, pre-exposure to CSE markedly enhanced NE-induced IL-8 production. As PAR2 acts as a receptor for NE, we next investigated the effect of CSE on PAR2 expression as a molecular mechanism for the increased IL-8 production induced by NE in CSE exposed cells. CSE, but not NE, increased the expression of PAR2 mRNA and surface membrane protein. Inhibition of p38 MAPK reduced PAR2 expression induced by CSE while inhibition of the ERK and Akt pathway had no effect. Consequently, p38 inhibition significantly abrogated CSE-induced enhancement of IL-8 production in NE-treated cells. Of note, we observed increased PAR2 levels in lung homogenates and lung epithelial cells from CSE-treated mice and from both smokers and patients with COPD. Taken together, these results suggest that CSE upregulates PAR2 in normal human bronchial epithelial cells, thereby enhancing the inflammatory response to NE.

## Lung disease: How cigarette smoking enhances lung inflammation

A study of the effect of cigarette smoke extract (CSE) on lung lining cells clarifies the interaction between CSE and an enzyme implicated in causing chronic obstructive pulmonary disease (COPD). The enzyme elastase is released by neutrophils and is known to induce inflammation, which causes lung irritation in COPD. Chul-Gyu Yoo and colleagues at Seoul National University in South Korea exposed cultured human lung cells to CSE. This exposure activated specific molecular signaling processes by which the elastase enzyme-induced inflammation was enhanced. Of particular note was an increase in the levels of a cell surface receptor protein in CSE-treated cells. This protein was increased in lungs of smokers and COPD patients. The research helps to understand the role of cigarette smoking in the pathogenesis of COPD.

## Introduction

Chronic obstructive pulmonary disease (COPD) is a progressive disease characterized by the presence of airflow limitation resulting from airway inflammation, airway remodeling, and parenchymal destruction. Cigarette smoke (CS) is the main known risk factor for the development of COPD. Although evidence-based data from experimental studies indicate that inflammatory cell-derived proteases are one of the major mediators^1^, the exact mechanism by which CS induces COPD development remains uncertain.

Neutrophils are one of the key inflammatory cells involved in this abnormal inflammatory response, which is associated with mucous metaplasia in chronic bronchitis and lung destruction in emphysema^2^. Neutrophils secrete various proteases, such as neutrophil elastase (NE), which can degrade most of the components of the pulmonary extracellular matrix and plays a crucial role in lung destruction in emphysema^3^. An important chemoattractant for neutrophils is IL-8 (CXCL8), which can be released by activated epithelial cells and other immune cells^4^.

Recently, proteinase-activated receptors (PARs) have been implicated in this process^5^. PARs are G-protein-coupled receptors and to date, PAR1, PAR2, PAR3, and PAR4 have been identified. PAR2 is expressed in human lung epithelial cells, airway smooth muscle cells, endothelial cells, human mast cells, macrophages and neutrophils^6,7^. Previous studies have shown different regulations of PAR expression according to inflammatory mediators and cell types. In endothelial cells, oxidative stress upregulates PAR2^8^, and the upregulation of PAR2 is mediated by p38 MAPK^9^. In pulmonary fibroblasts, PAR2 was stimulated by the profibrotic growth factors platelet-derived growth factor and transforming growth factor-β1^10^. Although the distribution of PAR2 and its response to inflammatory mediators have been studied, its function in bronchial epithelial cells and its response to cigarette smoke extract (CSE) are unclear. Both inflammation and protease-antiprotease imbalance interact with each other in the pathogenesis of COPD, and the inhibition of its pathways are an appealing approach for therapeutic interventions to break the vicious cycle. The objective of this study was to elucidate the effect of CSE on the NE-induced inflammatory response and its molecular mechanism in bronchial epithelial cells.

## Materials and methods

### Cells and reagents

Normal human bronchial epithelial cells (BEAS-2B) were maintained in defined keratinocyte serum-free medium (GIBCO by Life Technologies, Grand Island, NY, USA) at 37 °C under 5% CO_2_. Human sputum NE was purchased from Elastin Products Co. (Owensville, MO, USA). NE was dissolved in a solution of 50% glycerol and 50% 0.02 M NaOAc (pH 5). U0126 (an inhibitor of MEK1/2), LY294002 (an inhibitor of PI3K), and SB203580 (an inhibitor of p38 MAPK) were purchased from Cell Signaling (Danvers, MA, USA). Rabbit polyclonal anti-phospho-p44/42 MAPK (Thr202/Tyr204) (p-ERK), anti-phospho-Akt (Ser473) (p-Akt), and rabbit monoclonal anti-phospho-p38 MAPK (Thr180/Tyr182) (p-p38) antibodies were obtained from Cell Signaling. Mouse monoclonal anti-PAR2, anti-Hsp90, and goat polyclonal anti-GAPDH antibodies were obtained from Santa Cruz Biotechnology (Santa Cruz, CA, USA). Alexa Fluor 488 donkey anti-goat antibody and Hoechst33342 were purchased from Thermo Fisher Scientific (Waltham, MA, USA).

### Preparation of CSE

CSE was prepared as described in previous studies^11,12^. Commercial cigarettes (THIS; 84 mm long with a diameter of 8 mm), purchased from Korea Tomorrow & Global Corp. (Daejeon, Republic of Korea) were smoked continuously using a bottle system connected to a vacuum machine. The smoke from 20 cigarettes was bubbled in 60 ml of PBS (GIBCO). The large insoluble particles contained in the resulting suspension were removed by filtering the solution through a 0.22 μm filter.

### Protein extraction and western blot analysis

Total cellular proteins were extracted using 1X cell lysis buffer (Cell Signaling). Membrane proteins were isolated using a membrane protein extraction kit (Thermo Fisher Scientific). Frozen lung tissues (SNUH IRB Number: H-1309-073-521) were homogenized in tissue extraction buffer (Life Technologies) containing a protease inhibitor cocktail (Sigma-Aldrich, St. Louis, MO, USA) and a phosphatase inhibitor cocktail (Sigma-Aldrich). Protein concentration was measured using the Bradford protein assay according to the manufacturer’s instructions (Bio-Rad, Hercules, CA, USA). Proteins were resolved by 4–12% SDS-polyacrylamide gel electrophoresis (SDS-PAGE) and were transferred to nitrocellulose membranes. The membranes were blocked with 5% skim milk blocking buffer for 1 h before being incubated overnight at 4 °C with primary antibodies in blocking buffer. The membranes were washed with washing buffer three times and incubated with secondary antibodies for 1 h. After successive washes, the membranes were developed using the SuperSignal West Pico Chemiluminescent kit (Thermo Fisher Scientific).

### Multiplex bead assay

IL-8 levels in culture supernatants were determined using a commercially available Bio-Plex Pro^TM^ cytokine assay kit (Bio-Rad) according to the manufacturer’s instructions.

### Transfection of siRNA

Transfection of PAR2 siRNAs and control siRNAs was performed using the Neon Transfection System (Thermo Fisher Scientific) according to the manufacturer’s specifications. After 48 h, the cells were used in experiments.

### Real-time PCR

Total RNA was isolated using the RNeasy kit (Qiagen, Hilden, Germany). cDNA was synthesized from 1 μg of total RNA using the Reverse Transcription system (Promega, Madison, WI, USA). PCR amplification was performed with a 2X TaqMan gene expression master mix (Applied Biosystems, Foster City, CA, USA). The primer information is as follows: PAR2 (Hs00608346_m1) and GAPDH (Hs99999905_m1). The primers were obtained from Applied Biosystems.

### Immunofluorescence staining of PAR2

Cells grown in 35 mm dishes in the presence or absence of CSE for 24 h were fixed in methanol and incubated with rabbit polyclonal anti-PAR2 antibody diluted 1:100 in 3% BSA for 24 h. The cells were subsequently incubated with Alexa Fluor 488 donkey anti-goat antibody diluted 1:100 in 3% BSA for 30 min. After successive washes, the cells were analyzed under a fluorescence microscope (Nikon ECLIPSE TE300, Nikon Corporation, Tokyo, Japan).

### Intratracheal administration of CSE

Female 8-week-old C57BL/6 wild-type (WT) mice were purchased from OrientBio (Kapyong, Korea). Animal experiments were approved by the Institutional Animal Care and Use Committee (number 15-0121-S1A0(2)) of Seoul National University Hospital, Seoul, Korea. C57BL/6WT mice were anaesthetized and instilled intratracheally with vehicle or 100 μl CSE. CSE was instilled once a week for 8 weeks. Four mice were used in each group. The mice were sacrificed at week 8 after the first instillation to isolate the lungs.

### Immunohistochemistry

Lung tissues were fixed, embedded, cut and placed on slides using the Discovery XT automated immunohistochemistry stainer (Ventana Medical Systems, Inc., Tucson, AZ, USA). Tissue sections were deparaffinized and rehydrated. Cell conditioning 1 (CC1) standard (pH 8.4 buffer containing Tris/Borate/EDTA) was used for antigen retrieval. The sections were incubated with rabbit polyclonal anti-PAR2 antibody for 32 min at 37 °C, washed, and incubated with a secondary antibody for 20 min at 37 °C. After successive washes, slides were incubated with 3, 3-diaminobenzidine (DAB) H_2_O_2_ substrate for 8 min at 37 °C, followed by hematoxylin and bluing reagent counterstain. Stained cells were observed under a microscope (EVOS XL Core Cell Imaging System, Thermo Fisher Scientific).

### Statistical analysis

Statistical analysis was performed using GraphPad software. Data were analyzed using a two-tailed unpaired *t* test or Mann–Whitney *U* test, as appropriate, to determine statistical significance. Data from in vitro cell experiments represent the mean ± SD. Data from the experiments using mouse and human lung tissues are expressed as the mean ± SE. A *p*-value of <0.05 was considered significant.

## Results

### NE increases IL-8 production via PAR2-mediated activation of ERK

**Fig. 1:**
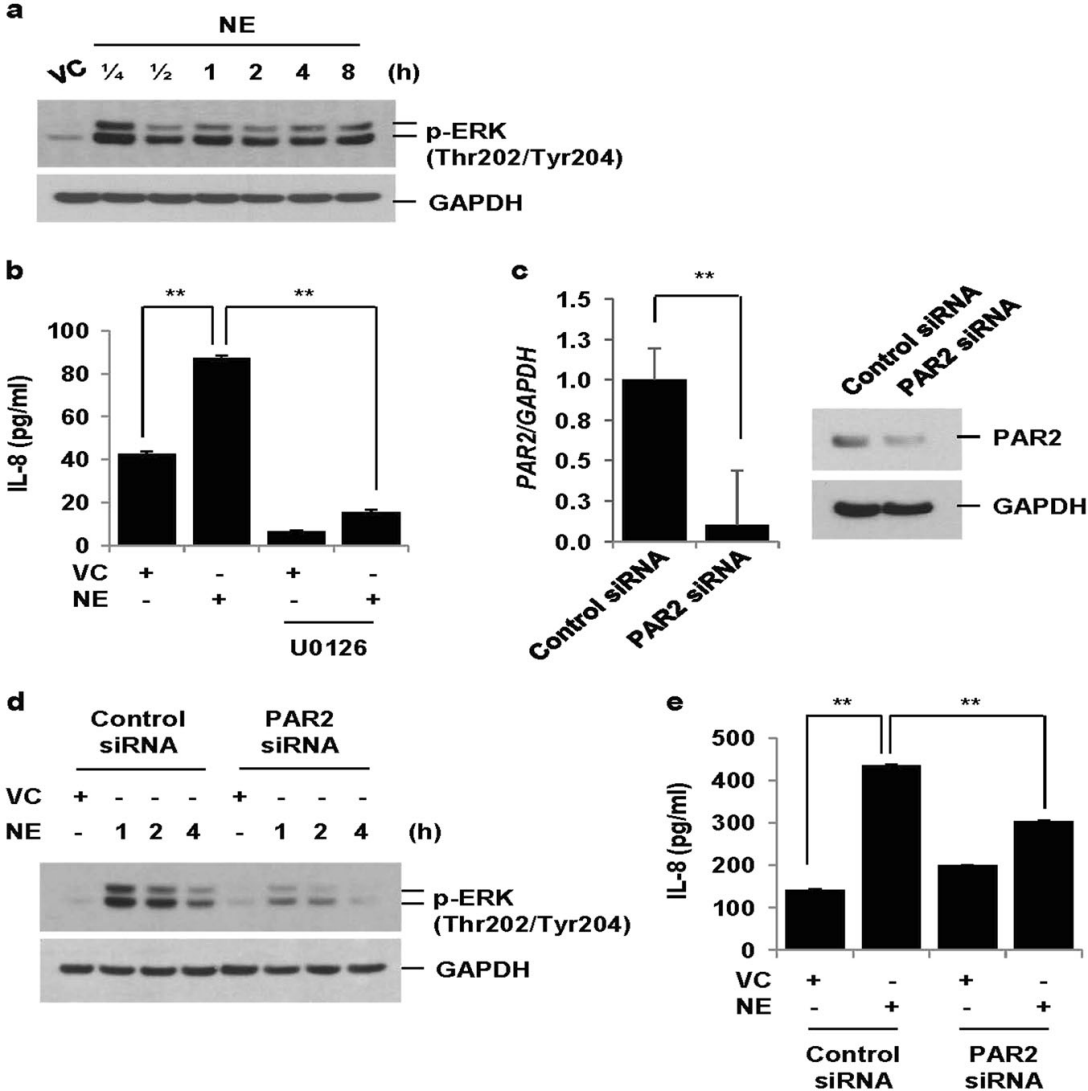
NE increases IL-8 production via PAR2-mediated activation of ERK. **a** BEAS-2B cells were treated with vehicle control (VC) or NE (1 U/ml) for the indicated times. Total cellular extracts were subjected to western blot analysis for p-ERK and GAPDH. **b** Cells were pre-treated with an MEK inhibitor (U0126, 20 μM) for 1 h and then stimulated with VC or NE (1 U/ml) for 24 h. Levels of IL-8 in cell supernatants were measured by multiplex bead assay. Data represent the mean ± SD; **P < 0.05. **c**–**e** BEAS-2B cells were transiently transfected with control siRNAs or PAR2 siRNAs using a Neon electroporation kit. Forty-eight hours after transfection, cells were stimulated with VC or NE for 1, 2, 4 h (**d**) or 24 h (**e**). The expression of PAR2 was measured by quantitative real-time PCR. Data were normalized to the expression of GAPDH. Data represent the mean ± SD; **P < 0.05. Total cellular extracts were subjected to western blot analysis for PAR2, p-ERK and GAPDH. IL-8 concentrations in culture media were measured by multiplex bead assay. Data represent the mean ± SD; **P < 0.05

NE activated ERK 15 min after treatment, which persisted for 8 h (Fig. 1a). NE also increased IL-8 production, which was blocked by an MEK inhibitor (U0126), suggesting that NE-induced IL-8 production is dependent on ERK activation (Fig. 1b). To evaluate whether PAR2 is involved in NE-induced ERK activation and subsequent IL-8 production, cells were transiently transfected with control siRNAs and PAR2 siRNAs. The mRNA and protein expressions of PAR2 were significantly decreased in PAR2 siRNA-transfected cells (Fig. 1c). Knockdown of PAR2 significantly suppressed NE-induced ERK activation and IL-8 production (Fig. 1d, e). These data suggest that NE-induced IL-8 production is dependent on PAR2-mediated ERK activation in lung epithelial cells.

### Pre-treatment with CSE enhances NE-induced IL-8 production via PAR2

**Fig. 2:**
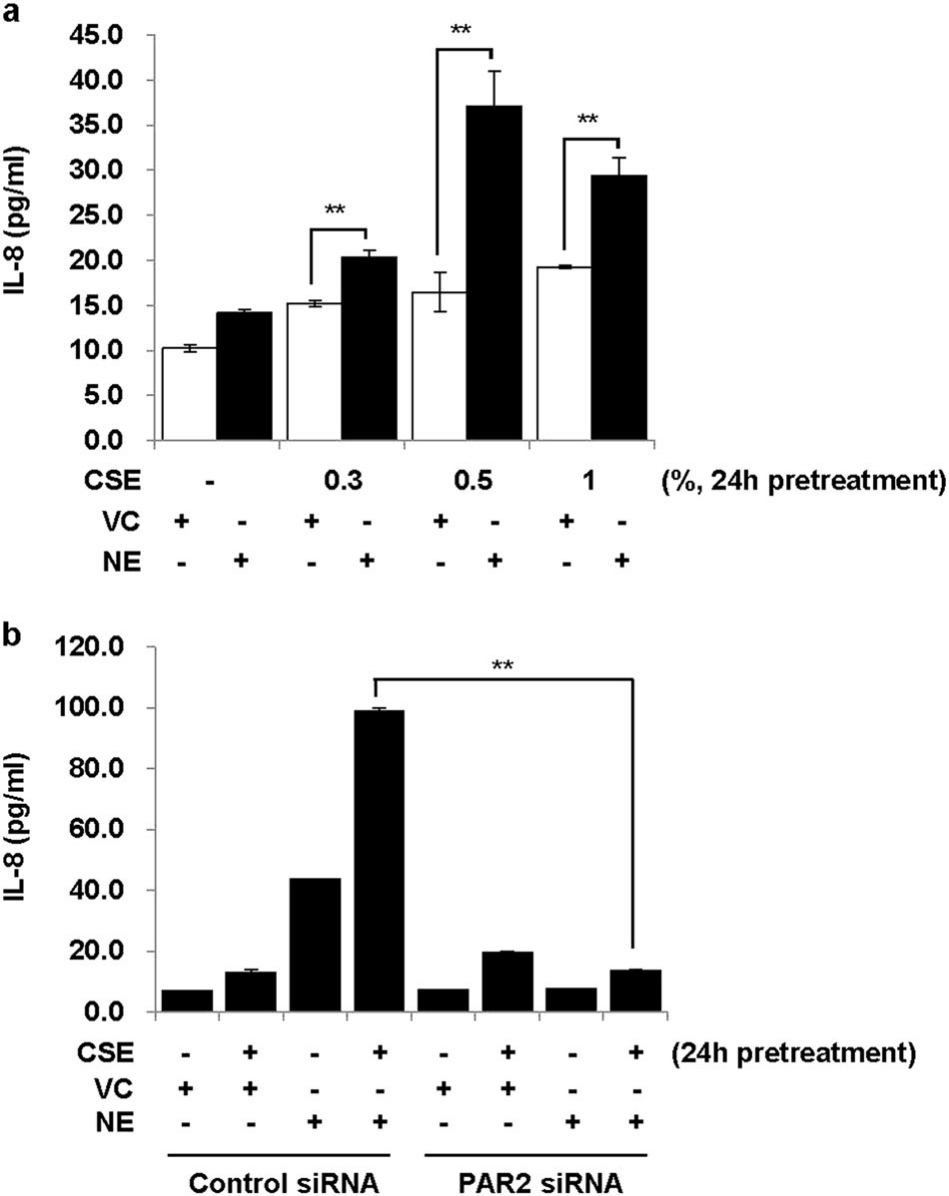
Pre-treatment with CSE enhances NE-induced IL-8 production via PAR2. **a** BEAS-2B cells were pre-treated with CSE (0.3, 0.5, and 1%) for 24 h and then stimulated with VC or NE (1 U/ml) for 24 h in the absence of CSE. **b** Cells were transiently transfected with control siRNAs or PAR2 siRNAs. Forty-eight hours after transfection, cells were pre-treated with CSE (1%) for 24 h and then stimulated with VC or NE (1 U/ml) for 24 h in the absence of CSE. IL-8 concentrations in cell supernatants were measured by multiplex bead assay. Data represent the mean ± SD; **P < 0.05

We next assessed the effect of CSE pretreatment on NE-induced IL-8 production. While pretreatment with CSE alone for 24 h only slightly increased IL-8 secretion, CSE enhanced NE-induced IL-8 production (Fig. 2a). PAR2 knockdown using siRNAs completely abrogated both NE-induced IL-8 production and CSE-induced enhancement of IL-8 release by NE (Fig. 2b). In contrast, a small increase in IL-8 production induced by CSE was not affected by knockdown of PAR2. These results suggest that NE-induced IL-8 production is enhanced by CSE pretreatment via PAR2.

### CSE, but not NE, upregulates PAR2 expression

**Fig. 3:**
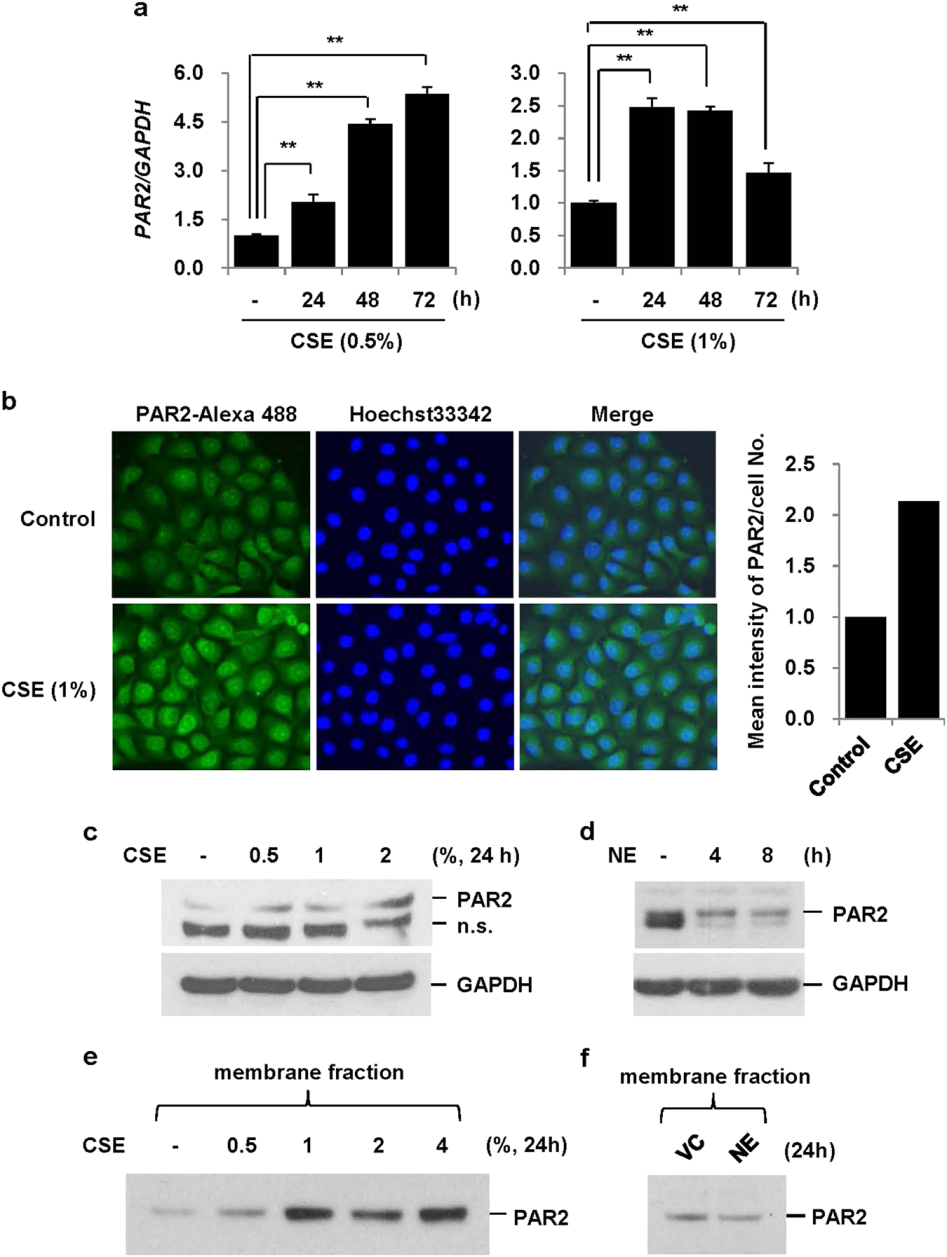
CSE, but not NE, upregulates PAR2 expression. **a** BEAS-2B cells were treated with CSE (0.5 and 1%) for the indicated times. The expression of PAR2 was measured by quantitative real-time PCR. Data were normalized to the expression of GAPDH. Data represent the mean ± SD; **P < 0.05. **b** Cells were stimulated with CSE (1%) for 24 h. The cells were fixed and permeabilized for 10 min. Immunofluorescent staining of PAR2 was performed using an anti-PAR2 antibody, followed by an Alexa Fluor 488 antibody. Cells were analyzed using an ECLIPSE TE300 (Nikon) fluorescence microscope. The mean intensity of PAR2 per cell number was determined using ImageJ. **c**, **d** BEAS-2B cells were treated with CSE (0.5, 1, and 2%) or NE (1 U/ml) for the indicated times. Total cell lysates were extracted and subjected to western blot analysis for PAR2 and GAPDH. **e**, **f** Cells were treated with CSE (0.5, 1, 2, and 4%) or NE (1 U/ml) for 24 h. The membrane fraction was isolated and then subjected to western blot analysis for PAR2 using an antibody to detect the N-terminal extracellular domain of PAR2. Results are representative of three independent experiments

As NE-induced IL-8 production is mediated through PAR2, we hypothesized that its enhancement by CSE pretreatment might be due to upregulation of PAR2 expression. Both the PAR2 mRNA and protein expression were significantly increased when BEAS-2B cells were treated with CSE (0.5, 1, and 2%) (Fig. 3a–c). In contrast, NE slightly decreased PAR2 expression (Fig. 3d). To determine if PAR2 expression increased at the membrane level, the membrane fraction was isolated and then subjected to western blot analysis using an antibody to detect the N-terminal extracellular domain of PAR2. PAR2 expression increased after CSE treatment in the membrane fraction (Fig. 3e) but did not increase after NE treatment (Fig. 3f). This finding suggests that upregulation of PAR2 on the membrane surface, which acts as a receptor for NE, might be associated with enhanced NE-induced IL-8 production after CSE pretreatment.

### CSE-induced upregulation of PAR2 is dependent on p38 activation

**Fig. 4:**
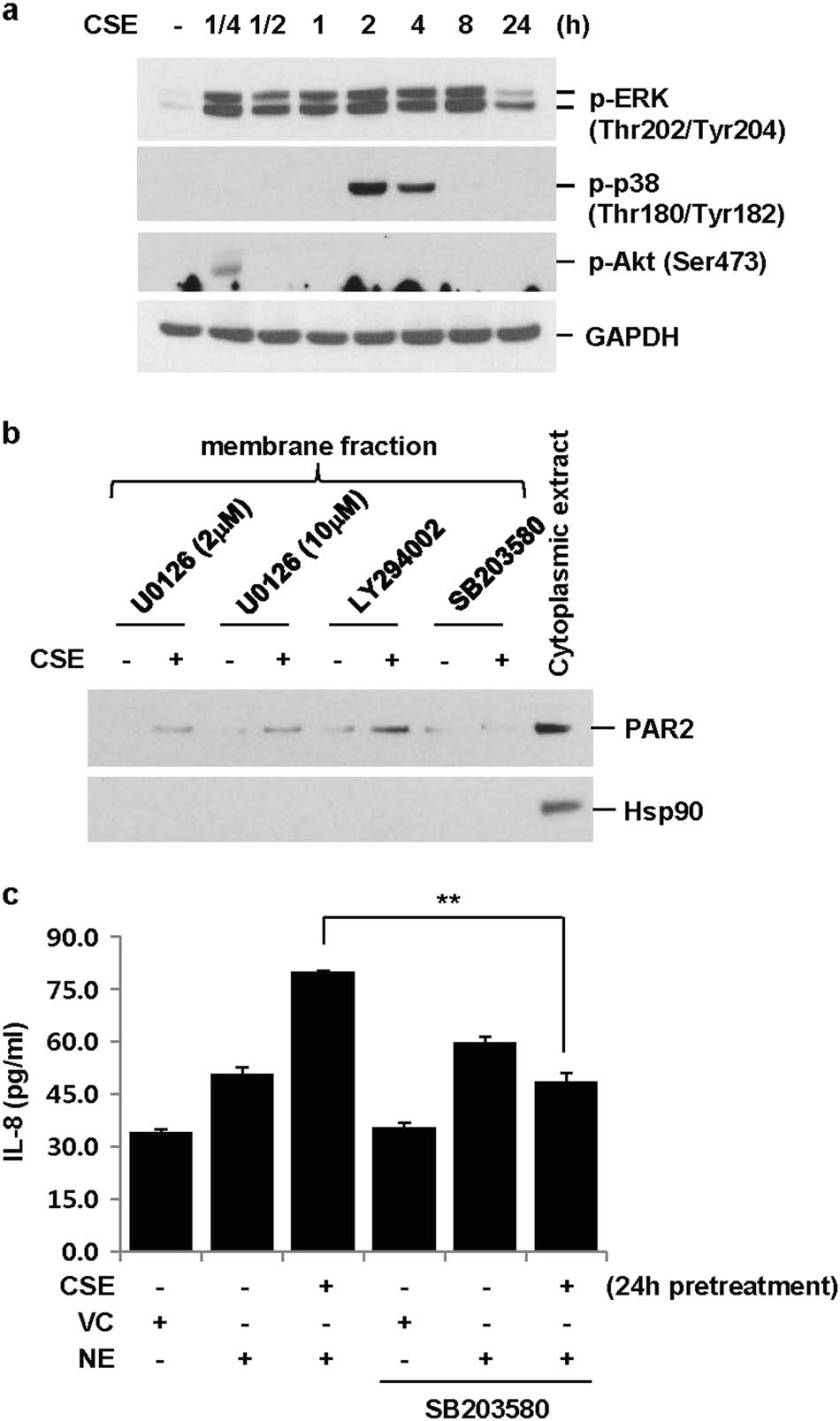
CSE-induced upregulation of PAR2 is dependent on p38 activation. **a** BEAS-2B cells were treated with CSE (1%) for the indicated times. Total cellular extracts were subjected to western blot analysis for p-ERK, p-p38, p-Akt, and GAPDH. **b** Cells were pre-treated with an MEK inhibitor (U0126, 2 or 10 μM), PI3K/Akt inhibitor (LY294002, 10 μM), or p38 inhibitor (SB203580, 10 μM) for 2 h and then stimulated with CSE for 24 h in the presence of U0126, LY294002, or SB203580. Membrane and cytoplasmic proteins were extracted and subjected to western blot analysis for PAR2 and heat shock protein 90 (Hsp90). **c** BEAS-2B cells were pre-treated with SB203580 (10 μM) for 2 h and then stimulated with CSE (1%) in the presence or absence of SB203580 for 24 h. The cells were stimulated with VC or NE (1 U/ml) for 24 h in the absence of SB203580 and CSE. IL-8 concentrations in cell supernatants were measured by multiplex bead assay. Data represent the mean ± SD; **P < 0.05

We next evaluated how CSE upregulates PAR2. To investigate which pathway is involved in the CSE-induced upregulation of PAR2, we evaluated the activation of MAPK and PI3K/Akt pathways. CSE induced the phosphorylation of ERK, p38, and Akt (Fig. 4a). Interestingly, the p38 inhibitor (SB203580), but not the MEK inhibitor (U0126) or PI3K/Akt inhibitor (LY294002), significantly inhibited upregulation of PAR2 in the membrane fraction (Fig. 4b). Consistent with these findings, p38 inhibition completely suppressed CSE-induced enhancement of IL-8 production in NE-treated cells, but it did not affect NE-induced IL-8 production (Fig. 4c).

### Increased level of PAR2 in lung homogenates and lung epithelial cells from CSE-instilled mice and from smokers and patients with COPD

**Fig. 5:**
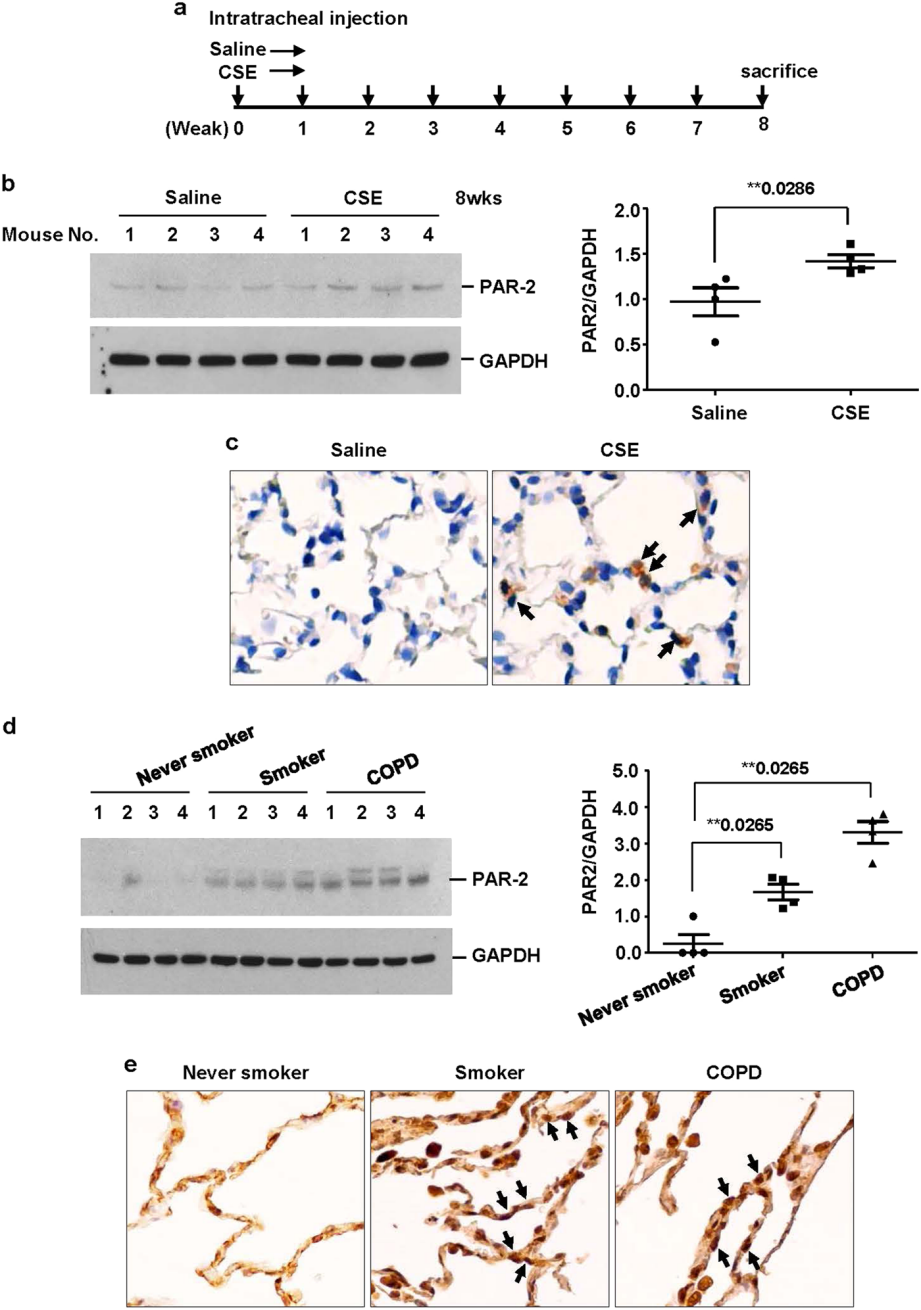
Increased level of PAR2 found in lung homogenates and lung epithelial cells from CSE-treated mice and from smokers and COPD patients. **a** Experimental protocols for a murine model of emphysema. C57BL/6 mice were intratracheally instilled with vehicle or CSE as described in the materials and methods section. Mice (*n* = 4 per group) were sacrificed at week 8 after first instillation to isolate lungs. **b** Lung homogenates from mice were subjected to western blot analysis for PAR2 and GAPDH. Gel data were quantified using Scion image densitometry (right panel). Data represent the mean ± SE; **P < 0.05. **c** PAR2 immunohistochemistry in the lung tissues from mice. PAR2 was increased in lung epithelial cells of CSE-treated mice (arrows). **d** Total lung lysates obtained from normal (*n* = 4), smokers (*n* = 4), and COPD patients (*n* = 4) (**c**) were subjected to western blot analysis for PAR2 and GAPDH. Gel data were quantified (right panel). Data represent the mean ± SE; **P < 0.05. **e** PAR2 immunohistochemistry in the lung tissues from human patients. Arrows identify the epithelial cells. Original magnifications, ×200

To examine the effect of CSE on PAR2 expression in mouse lung tissue, C57BL/6 mice were intratracheally instilled with saline or CSE as described in Fig. 5a. CSE increased the level of PAR2 expression in mouse lung tissues (Fig. 5b). Immunohistochemical staining for PAR2 in formalin-fixed and paraffin-embedded mouse lung tissues showed that PAR2 was increased in the lung epithelial cells of CSE-instilled mice (Fig. 5c). We next compared the expression level of PAR2 in lung tissues from normal (non-smokers), smokers, and COPD patients. PAR2 expression was significantly higher in lung extracts and the lung epithelial cells of smokers and COPD patients (Fig. 5d, e).

## Discussion

**Fig. 6:**
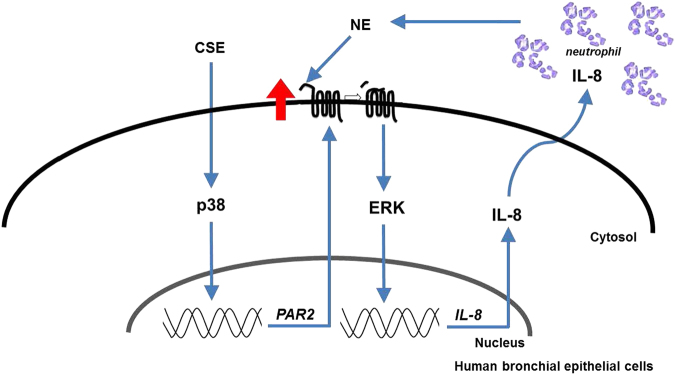
Schematic diagram of the cross-talk between CSE and NE. CSE increases IL-8 production in lung epithelial cells. Released IL-8 recruits neutrophils into the lungs. Neutrophils secrete several proteases including NE. NE induces IL-8 production via the PAR2-mediated activation of ERK. CSE upregulates PAR2 through a p38-dependent pathway, which enhances NE-induced IL-8 production. A vicious cycle causing continued neutrophil accumulation and inflammation exists

COPD is an inflammatory disorder and neutrophils are considered to play an integral role in the pathophysiology of COPD^13,14^. Experimental models of emphysema and individuals with genetic deficiency of alpha-1 antitrypsin provide strong evidence that NE is associated with alveolar wall destruction and mucus hypersecretion^15^. In addition, airway neutrophilic inflammation is associated with COPD disease progression, disease severity, and exacerbations^16,17^. One of the most important risk factor for COPD is cigarette smoking, and it is clear that smoking evokes inflammatory responses^1^, but how do they all work together in the pathogenesis of COPD? This study showed that NE induces IL-8 production in human bronchial epithelial cells via PAR2 and that IL-8 is enhanced by CSE through PAR2 upregulation (Fig. 6).

Serine proteases activate a family of G-protein–coupled receptors called PARs to mediate some of their effects^18^. Among the four PARs (PAR1, PAR2, PAR3, and PAR4), research on chronic lung disease has particularly focused on PAR2^6^. There have been many studies on PAR2 and the development of allergic airway inflammation^19,20^. Recent studies report an association of PAR2 activation with pulmonary fibrosis^21^. Additionally, PAR2 is expressed in the alveolar epithelial cells of idiopathic pulmonary fibrosis (IPF) patients, and the expression of PAR2 significantly correlates with the extent of honeycombing^22^, suggesting a role of PAR2 in the development of IPF. However, the role of PAR2 in the development of COPD has not yet been elucidated. Studies using human lung epithelial cells (A549 cells) showed that PAR2 agonists increased IL-8 production but showed conflicting results for PAR1 agonists^23,24^. In our previous study, pulmonary bronchial epithelial cells were treated with PAR1-activating peptide (PAR1-AP) and PAR2-AP and both showed increased IL-8 production. However, only PAR2-blocking peptide significantly suppressed NE-induced IL-8 production^25^. Knockdown of PAR2 also reduced IL-8 release in NE-treated cells. Similar findings have been shown in corneal^26^, gastric, and colonic epithelial cells^27^.

CS is the major environmental risk factor for COPD. CS is reported to induce production of oxidants from inflammatory cells. CS directly or indirectly, through oxidants, triggers chronic inflammation by releasing inflammatory cytokines, such as TNF-α^28^ and IL-8^12^. IL-8 secreted from bronchial epithelial cells by CS mediates the recruitment of neutrophils into the lung and thus further amplifies chronic inflammation. In this study, CSE slightly increased IL-8 production and pre-exposure of CSE enhanced NE-induced IL-8 production in lung epithelial cells. CSE itself not only induces inflammation but also primes cells to cause further inflammation. CSE upregulated PAR2 via the activation of the p38 MAP kinase pathway and CSE-induced upregulation of PAR2 mediated the enhancement of IL-8 production by NE. In addition, we showed that CSE upregulates PAR2 expression in lung tissues of a murine emphysema model. A previous study demonstrated that intratracheal administration of elastase increases CSE-induced IL-8 in bronchoalveolar lavage fluid and enhances CSE-induced emphysema in mice^12^. CSE-induced upregulation of PAR2 might contribute to the development and progression of emphysema in mice. Consistent with these findings, PAR2 protein was increased in lung homogenates and lung epithelial cells from smokers and COPD patients.

Cigarettes consist of at least 3800 chemical constituents. CS has more than 4000 chemicals and additives, many of which are pharmacologically active, toxic, and carcinogenic^29^. Major constituents of CS, such as tar, nicotine, carbon monoxide, nitrogen oxides, hydrogen cyanide, and metals, have been identified as the most likely to cause several diseases including COPD. Many of these components reportedly induce inflammation, oxidative stress, and apoptosis. As a result, lung damage leading to emphysema is induced. Among these components, cigarette tar can produce large amounts of hydrogen peroxide in aqueous extracts^30^. Hydrogen peroxide can upregulate PAR2 in keratinocytes^31^. However, it is not known which major component in CS (and CSE) increases PAR2. To identify the component, further detailed study is required.

In conclusion, our study reveals a cross-talk between oxidative stress (CSE) and a protease (elastase) in lung epithelial cells. The interactive action of CSE and elastase mediates further neutrophilic inflammation, consequently contributing to COPD pathogenesis.
